# Numerical investigation of the sternoclavicular joint modeling technique for improving the surgical treatment of pectus excavatum

**DOI:** 10.1038/s41598-020-64482-7

**Published:** 2020-04-30

**Authors:** Beop-Yong Lim, Youngwoong Kim, Hoseok I, Chi-Seung Lee

**Affiliations:** 1 Department of Biomedical Engineering, Gradate School, Pusan National University, Busan, 49241 Republic of Korea; 2 University Research Park of Pusan National University, Busan, 49241 Republic of Korea; 3 Department of Thoracic and Cardiovascular Surgery, Trauma Center, Ulsan University Hospital, University of Ulsan College of Medicine, Ulsan, 44033 Republic of Korea; 4 Department of Thoracic and Cardiovascular Surgery, School of Medicine, Pusan National University, Busan, 49241 Republic of Korea; 5 Biomedical Research Institute, Pusan National University Hospital, Busan, 49241 Republic of Korea; 6 Department of Convergence Medicine, School of Medicine, Pusan National University, Busan, 49241 Republic of Korea; 7 Department of Biomedical Engineering, School of Medicine, Pusan National University, Busan, 49241 Republic of Korea

**Keywords:** Biomedical engineering, Adaptive clinical trial

## Abstract

It is now common to perform the Nuss procedure as a surgical treatment for pectus excavatum. As several types of detailed surgical methods exist as part of the Nuss procedure, studies are currently being conducted to verify their relative superiority via computerized biomechanical methods. However, no studies have considered the influence of sternoclavicular joints on the simulations of the Nuss procedure. Accordingly, this study aims to demonstrate the influence of these joints by comparing the clinical data with the finite element analysis data. Scenarios were set by classifying the movement of the joints based on the constraints of translation and rotation in the coordinate plane. The analyses were performed by applying the set scenarios to the constructed finite element model of a chest wall. The sternal displacement, Haller index, and equivalent stress were obtained from the analysis, and the data were compared with the data of the postoperative patient. When the translation of the anterior direction on the chest wall was constrained, the result obtained thereof was found to be similar to those obtained in the actual surgery. It is suggested that more accurate results can be obtained if the influence of the sternoclavicular joints is considered.

## Introduction

Pectus excavatum (PE) is a sharp posterior curve of the body of the sternum, i.e., a deformity of the chest wall with its greatest depth just prior to the junction with the xiphoid^1^. It is one of the common congenital anomalies that occurs in approximately 2–3 out of 1,000 newborns^2^. Among the many surgical methods existing for treating PE, the Nuss procedure is most commonly used. This procedure is performed on the soft and pliable costal cartilages and ribs of a child’s chest wall without performing rib cartridge incisions, resections, or sternal osteotomies. By inserting a concave pectus bar through a small lateral thoracic incision, serious PEs in prepubertal patients can be corrected^3^. As a result of several clinical studies performed on numerous patients, this treatment has been verified as a safe and effective surgical procedure with low recurrence rates and high patient satisfaction^4,5^.

PE patients are typically segregated into symmetric and asymmetric types via computed tomography (CT) scans, and each of these types can be further subdivided into depression, broad, flat, and eccentric types depending on the location and asymmetric shape of the sternum^6^. If the PE type has a complicated shape, detailed surgical methods such as fixation, inserted location, and number of pectus bars in the Nuss procedure should be carefully considered and applied^7^. The use of inappropriate surgical methods may result in the use of trial and error, which requires the reinsertion of the pectus bar during surgery, thereby resulting in complications and prolonged operative times. In order to avoid this problem, surgical methods have been evaluated in detail in various clinical studies, but the relative benefits between each surgical method are uncertain. Therefore, additional studies have recently been conducted to predict the form of the postoperative chest wall using a finite element (FE) analysis^8–10^. This computerized biomechanical verification of the Nuss procedure is performed by comparing the actual surgery data with the output mechanical values according to the predicted form after inserting the pectus bar into the developed chest wall of the FE model^11,12^. However, several problems are associated with the FE method, one of which is related to the influence of the sternoclavicular (SC) joints.

As the pectus bar is raised, the manubrium and xiphoid process of the sternum are also raised. However, the levels of elevation of these two positions can be different when the actual Nuss procedure is performed. The xiphoid process can move freely with the elevation of the pectus bar, but the movement of the manubrium is limited as it is connected to the clavicles. The connection points are SC joints, and the resistance to the sternum is increased or decreased owing to their movability. Currently, studies performed to verify the Nuss procedure via the FE method do not consider the resistance of these SC joints. However, this is a significant factor to consider when examining the mechanical behavior of the chest wall, and it is clinically recognized that this influences the elevation of the sternum. For example, despite the significant influence of the SC joints during the actual surgery of a funnel chest with a long depression, there currently exists no FE method for evaluating these joints. Therefore, by verifying the influence of the SC joints, we will be able to conduct a more accurate evaluation of the Nuss procedure.

After geometrically classifying the mechanical behavior of the SC joints in this study, the FE analysis of the Nuss procedure was performed by applying computerized biomechanical methods, and several scenarios were established. The FE analysis was performed to compare the calculated results of the displacement, equivalent stress of the chest wall, and pectus bar with the chest wall cross-sectional shape after the Nuss procedure was performed for patients with PEs. Previous PE studies have not considered the influence of the SC joints; therefore, this study aims to verify this influence via an FE analysis. We assessed the similarity between clinical data and the results of the FE analysis and studied the manner by which joint affects the reliability of the PE analysis.

## Methods

A series of steps should be followed to obtain a computational simulation of the Nuss procedure. First, the ranges for simulating the chest wall, surgical assumptions, and analysis methods should be defined. This step is basic to the study because it affects the development of the FE model and the efficacy of the analysis. The designs of the desired anatomical shapes derived from the applied mechanical analysis are the required next steps for the development of the FE model. The model should be precisely constructed while considering the location of the applied external force and the different properties of the materials in the contact range. Finally, the simulations should be performed based on the selection of the FE analysis method and several scenarios. These analyses results can be compared with patient clinical data and validated systematically.

A 15-year-old male patient with PE of the symmetric and depression type, with clear pre- and post-surgical medical image data. He received the Nuss procedure and visited the Pusan National University Hospital, was selected from whom to obtain clinical data for comparison with the FE analysis data. This study was performed in accordance with the relevant guidelines and regulations. The parents of the patient provided written informed consent for the patient to participate in the study and permitted access to the patient’s medical images. In addition, this study has been approved by the Institute Review Board (IRB) of the Pusan National University Hospital (IRB No. 1910-017-084).

### Development of FE model

The body parts of the patient required to be simulated prior to the implementation of the Nuss procedure in a 3D computer system can be divided into the tissues of the chest wall and the inserted medical device, i.e., the pectus bar. The tissues that are mainly affected by the behavior of the chest wall during surgery are selected first for modelling before the 3D modeling of the chest wall is performed. As the sternum of the chest wall showed anterior and posterior movement in the sagittal plane, the sternum, ribs, and costal cartilage were selected for modelling.

The CT-scan image in the medical data of the patient was imported using 3D medical image processing software (Mimics Innovation Suite 22.0, Materialise, Leuven, Belgium). After the selected tissues were separated and marked, their 3D models were constructed. The 3D model should comprise several solids, and its surface was smoothed for achieving accuracy and convergence via wrapping and smoothing^13^.

**Figure 1:**
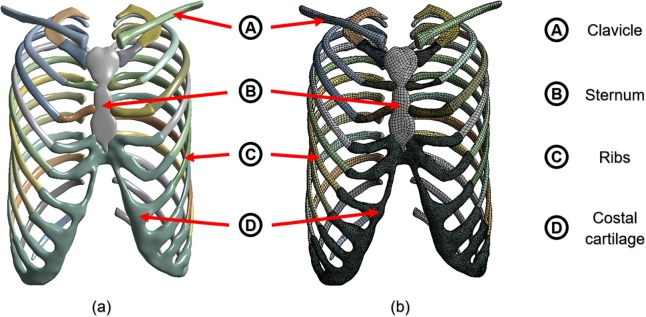
FE model was constructed using the sternum, ribs, clavicle, and costal cartilage of the chest wall: **(a)** 3D model with surface treatment after requisite tissues was extracted from CT image; **(b)** Mesh of FE chest model.

As the shapes of the tissues (sternum, ribs, clavicle, and costal cartilage) were complicated, the solid surfaces and curves were also complicated in the constructed 3D model. Accordingly, appropriate tetrahedral and hexahedral elements were incorporated in the model. In addition, the element sizes were reduced to include locations where the external forces were applied and where they came in contact with other objects. Figure 1 shows the results obtained for each of the above processes.

It was important to manufacture the pectus bar medical device using patient-specific geometries. We predicted the postoperative normal curve from the chest wall section of the preoperative patient in a manner similar to that of the existing surgical procedure. A design of the pectus bar was adopted that was substituted for this curvature^14^.

**Figure 2:**
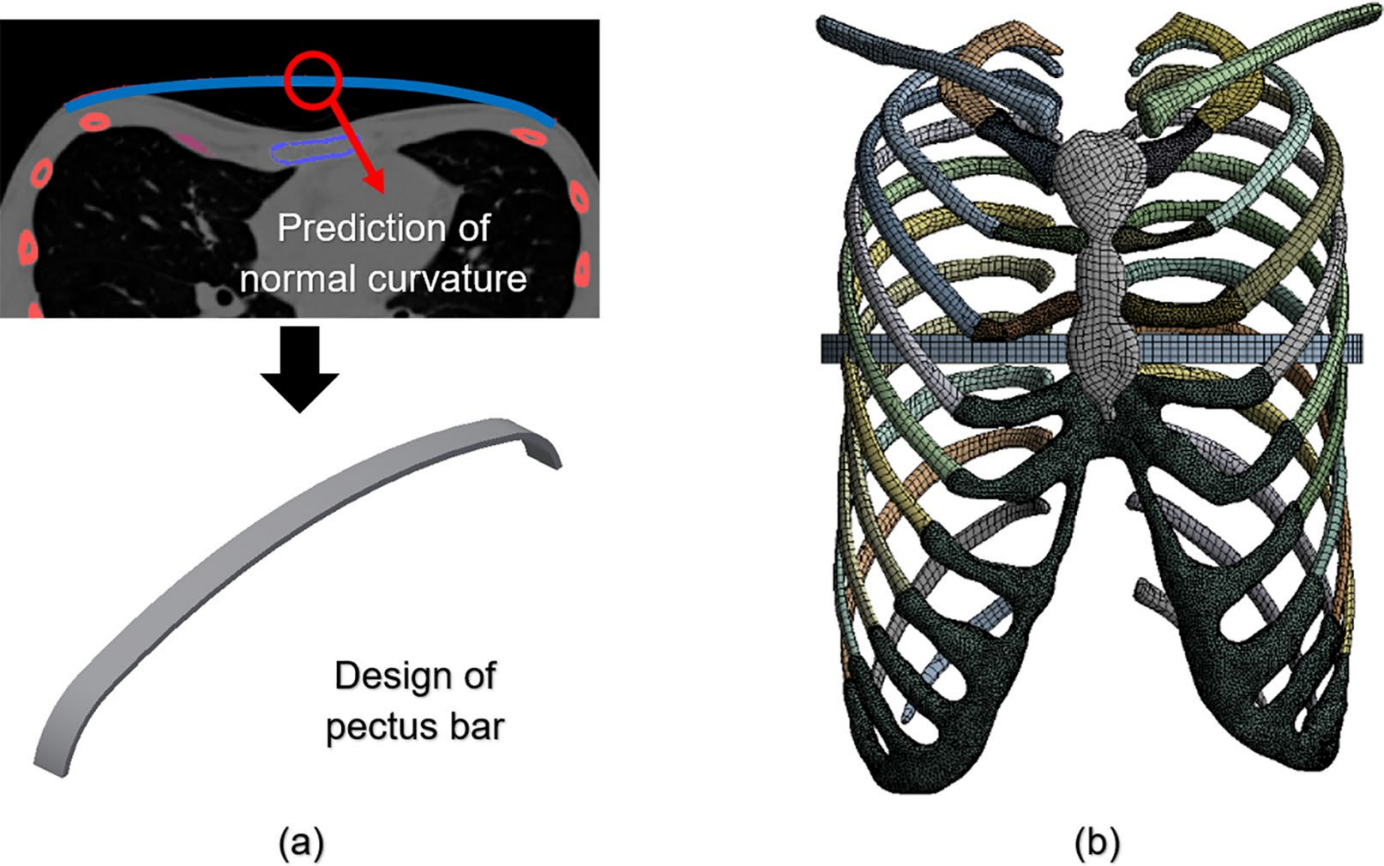
Principle schematics of the pectus bar: **(a)** Design of pectus bar for predicting postoperative curve using CT scan; **(b)** Final FE model with pectus bar inserted in the chest wall.

The curve was determined by the prediction of the postoperative chest wall and was based on the selection of the insertion point of the pectus bar using CT images. As the predicted curve applicable to the design of the pectus bar should be simplified, a curvature including no more than four curves was constructed. Inventor 2019 (Autodesk, Mill Valley, USA) was used for the modeling of the pectus bar, which was designed based on the length and width dimensions of a straight bar applied to the patient. The curved bar obtained using the above predicted curvature was constructed as shown in Fig. 2(a). The designed bar comprised the use of a hexahedral element owing to its simple mechanical shape. The element size was the same as that of the sternum and ribs. Furthermore, the area that would be in contact with the chest wall comprised a denser material.

The relative position of the developed FE models should include the pectus bar inserted into the chest wall. The pectus bar insertion point was at the fourth intercostal section, the same location as the existing postoperative section. In addition, the upper part of the pectus bar was placed in the lower part of the sternum because the sternum must be raised by the pectus bar, thereby correcting the PE. Figure 2 (b) shows the combined FE model of the chest wall, and Table 1 shows the type and number of elements and nodes used in each part of the FE model.

**Table 1:** Characteristics of elements in the FE model of chest wall and pectus bar.

	Element type	Number of elements	Number of nodes
Sternum	Hexahedral solid	2,470	8,347
Ribs	Hexahedral solid	34,242	110,758
Clavicle	Hexahedral solid	3,378	10,647
Costal cartilage	Tetrahedral solid	94,594	172,097
Pectus bar	Hexahedral solid	280	2,333
Total		134,964	304,182

### Selection of the scenarios

For a complete analysis to be performed using the constructed FE model, the scenarios should be selected by determining conditions and variables suitable for this study. As described above, the significance of this study is to identify the effects of SC joints in the simulations of the chest wall, such that the movability of the SC joints becomes a major classification.

**Figure 3:**
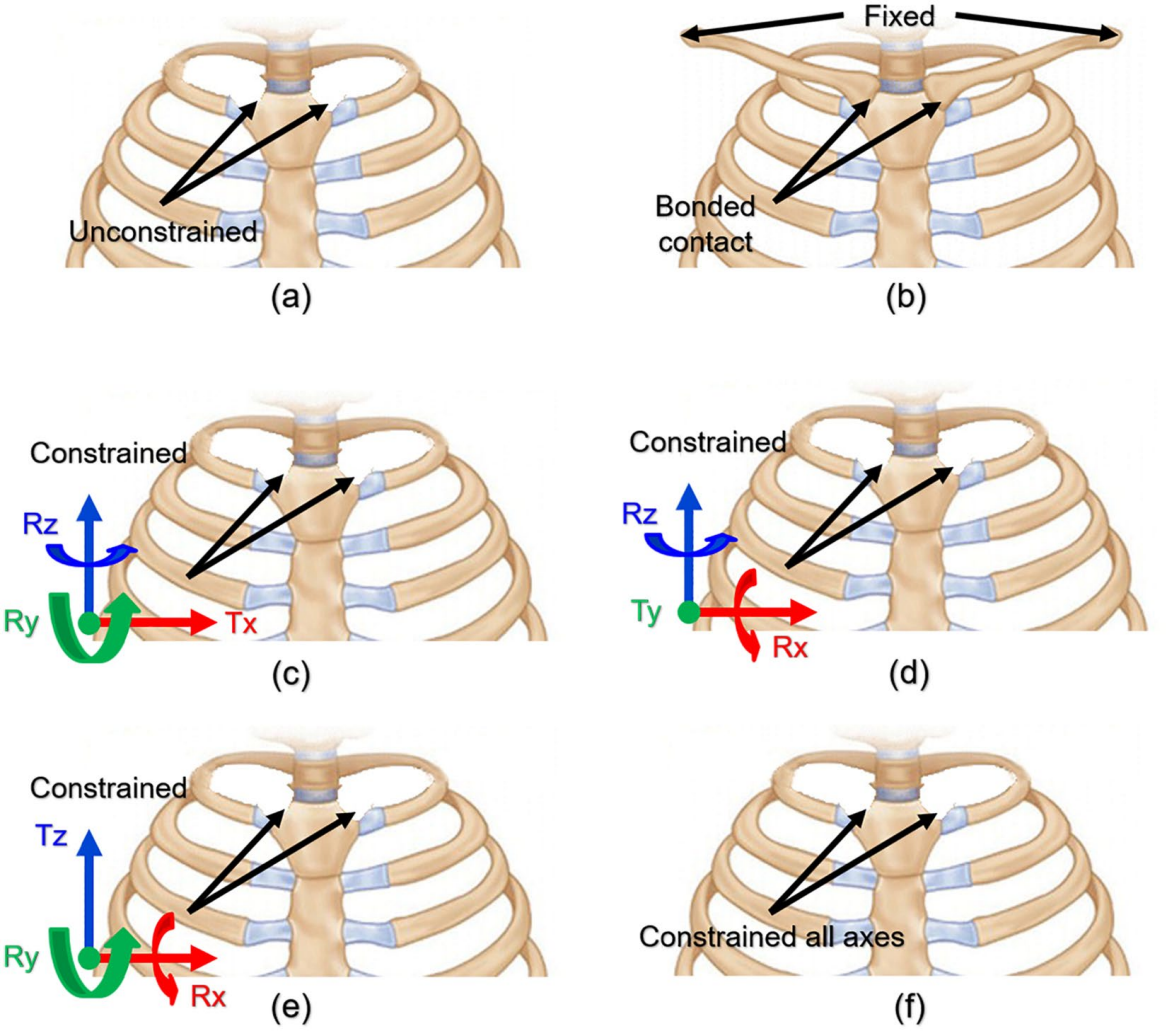
Visualization of constraints of SC joint behavior under different conditions: **(a)** All movements are allowed in scenario 1; **(b)** In scenario 2, FE model of the chest wall incorporates clavicles associated with sternum with constrained acromial ends; **(c)** In scenario 3, displacement is constrained along x-axis, and rotation is constrained along the other axes; **(d)** In scenario 4, displacement is constrained along y-axis, and rotation is constrained along the other axes; **(e)** In scenario 5, displacement is constrained along z-axis, and rotation is constrained along the other axes; **(f)** In scenario 6, all the axes are constrained such that there is no displacement or rotation.

Various combinations of SC joint movements exist based on the coordinate axis. We selected the following behaviors that can be moved according to the SC joint anatomical criteria. The movement of the SC joint is assumed to constrain the displacement about one axis, constrain the rotation of the other axes, and allow additional movements. Accordingly, scenarios wherein each of the x, y, and z axes are controlled should be considered. In addition, the conditions that constrained state all axes for comparison with the above axis behavior and free state such as studies without considering the SC joint are assumed. Meanwhile, the SC joints comprise articular sites for the clavicles on the manubrium and the sternal facets of the clavicles. Therefore, as clavicles have a major influence on the movements of the SC joints, it is possible to control the movements of the SC joints by constraining the acromial ends of the clavicles. This SC joint movement was included in the scenarios. All the scenarios described above are summarized in Fig. 3 and Table 2.

**Table 2:** Mathematical representation and detailed description for each selected scenario.

Scenario No.	Constraints	Detailed description
1	$Tx=Ty=Tz\ne 0\quad Rx=Ry=Rz\ne 0$	• Except for the clavicle, free behavior owing to there being no constraints on the SC joints
2	—	• Relationship between the clavicle and sternum was established. • Both acromial ends of the clavicle were fixed.
3	$Ty=Tz\ne 0,\,Tx=0\quad Ry=Rz=0,\,Rx\ne 0$	• SC joint’s x-axis translation and y- and z-axis rotation were constrained. • The remaining behavior was unrestricted.
4	$Tx=Tz\ne 0,\,Ty=0\quad Rx=Rz=0,\,Ry\ne 0$	• SC joint’s y-axis translation and x- and z-axis rotation were constrained. • The remaining behavior was unrestricted.
5	$Tx=Ty\ne 0,\,Tz=0\quad Rx=Ry=0,\,Rz\ne 0$	• SC joint’s z-axis translation and x- and y-axis rotation were constrained. • The remaining behavior was unrestricted.
6	$Tx=Ty=Tz=0\quad Rx=Ry=Rz=0$	• Except for the clavicle, the SC joints were constrained using a fixed condition.

Note: Tx, Ty, and Tz translate in the direction of each axis; Rx, Ry, and Rz rotate around each axis.

### Material properties of FE model

The tissues modeled in this study are the bone types of the sternum, ribs, clavicle, and costal cartilage. The respective properties of bone—which is divided into cortical and cancellous bone—should be applied, and each cortical bone thickness should also be applied separately. These were assumed to be constant for the convenience of modeling and analysis, and as the rib comprises several bones, it is divided into four parts according to its position. Therefore, each rib thickness was specified as manubrial (ribs 1–2), sternal (ribs 3–7), floating (ribs 8–10), and false (ribs 11–12). Table 3 shows the cortex thickness for each type of bone used to this study^15–17^.

**Table 3:** Cortex thickness of each bone type.

Bone type		Cortex thickness (mm)
Sternum		2.1
Ribs	(Manubrial)	0.685
	(Sternal)	0.7
	(Floating)	0.725
	(False)	0.685
Clavicle		3.4

As mentioned above, the bone types can be divided into cortical and cancellous bones, each with different material properties. However, it was confirmed by FE analysis of the clavicle that the material properties of the clavicle had little effect on the behavior of the chest wall (see Supplementary Table S1). Accordingly, the material properties of the clavicle were simplified in this study. In addition, the properties of the tissues could be designed to simulate those of the costal cartilage. The pectus bar material properties were selected on the basis of titanium^18–20^. In addition, the internal shape of all the materials was assumed to be homogeneous, and Young’s modulus and Poisson’s ratios were applied because only the movement within the elastic section was confirmed in the actual Nuss procedure. Table 4 summarizes the overall properties of the biomaterials and pectus bar.

**Table 4:** Material properties of biomaterial tissues and pectus bar.

Materials		Young’s modulus (MPa)	Poisson’s ratio
Sternum	(Cortical bone)	11,500	0.3
	(Cancellous bone)	40	0.45
Ribs	(Cortical bone)	5,000	0.3
	(Cancellous bone)	40	0.45
Clavicle		17,000	0.3
Costal cartilage		37.5	0.3
Pectus bar		200,000	0.3

### Setting of contact, displacement, and boundary conditions

As a condition for establishing the FE analysis, direct and indirect external forces and the conditions supporting them must be applied in the FE model. The external force on the chest wall in the Nuss procedure is the force applied when the pectus bar is raised. The force owing to the pectus bar depends on the raised height, and the determination of this displacement is critical. In this study, the conditions other than the constraints of the SC joint should be consistent with the actual surgical results. Therefore, the displacement was determined by confirming the anterior/posterior length of the chest wall before and after the actual surgery is performed. The difference in length before and after the surgery was 10 mm, and the height of the raised bar in the simulation was set as described above.

The sternum and ribs and the costal cartilage that connects them are fixed with no separation of these contacts. Similarly, the clavicle inserted in scenario 2 is also fixed. Accordingly, the contact conditions of the above tissues were set as those of bonded contacts to prevent their separation. The sternum is pushed upwards because of the raised pectus bar, and both objects are moved simultaneously with the same displacement. To simulate this case, the condition between the two objects was also set as a bonded contact, similar to that in the case of the actual surgery.

**Figure 4:**
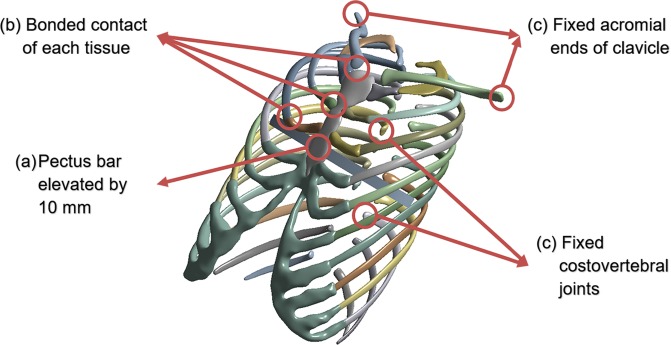
Contact, displacement, and boundary conditions were applied in the FE model: **(a)** Pectus bar was raised by 10 mm in the direction of the anterior sternum; **(b)** Contact conditions of multiple bones, costal cartilage, and pectus bar; **(c)** Boundary conditions set for the ribs and clavicles.

When an external force is applied to an object, a boundary condition should be used. In the Nuss procedure, the cervical and thoracic spines support the elevation of the sternum. The spine, being articulated with the ribs, transfers its supported force to the sternum, which is outside the scope of this study. Therefore, the costovertebral and costotransverse joints connecting the vertebrae and ribs were set as having fixed conditions to prevent rib displacement. Moreover, the acromial ends of the clavicle in scenario 2 were also fixed. The schematic in Fig. 4 describes these various set conditions.

## Results

ANSYS 2019 software (Ansys Inc., Canonsburg, USA) was used for conducting the FE analysis of each scenario, and the displacement of the chest wall shape and the equivalent bone stress were analyzed. In particular, the displacement scenario was determined using a high similarity through comparison with postoperative image data of an actual patient. The equivalent stress was used as an indicator of the difference between the two scenarios. In addition, the safety range of the chest wall was estimated by identifying the maximum equivalent stress.

### Displacement of two points in sternum of chest wall

**Figure 5:**
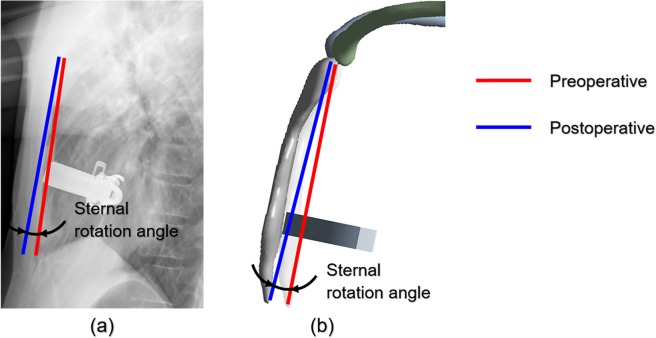
Example of measuring sternal rotational angle results of surgery and simulation. **(a)** Slope measurement of rotational angle of sternum using patient’s pre/postoperative data; **(b)** Rotational angle in the analysis of each scenario.

When the pectus bar is raised, the anterior and posterior width of the chest wall is increased, and sternal displacement occurs. At this time, the pectus bar is raised in the vertical direction of the anterior sternum plane, but the sternum does not move horizontally owing to the various tissues connected to the sternum. This results in varying displacements for the manubrium and xiphoid process. The points most influential in enabling this movement are the SC joints. Accordingly, a straight line was realized to connect the upper end of the manubrium with the lower end of the xiphoid process. As shown in Fig. 5, this straight line is divided into two straight lines before and after performing the surgery and simulation. The rotational angle for the results obtained after the simulation was derived to measure the angle between these two straight lines.

As a baseline for each scenario, the postoperative rotational angle was measured at 3.46° from the sagittal plane of the chest wall. The rotational angles of each scenario and the relative difference ratio between the postoperative values and the results of each scenario are listed in Table 5. This ratio was used to determine the similarity between the postoperative value and the scenarios and was expressed as the absolute value of the arithmetic ratio.

**Table 5:** Comparison of sternal rotational angles between actual surgery and simulation results.

	Postoperative	Scenario Number					
		1	2	3	4	5	6
Rotation angle (°)	3.46	1.00	1.28	0.99	4.76	0.62	5.40
Relative difference^a^ (%)	0	71.01	63.00	71.39	37.57	82.08	56.07

^a^Quantification of the relative difference in rotation angles for each scenario based on postoperative rotation angle.

Scenarios similar to the postoperative rotational angle can be identified in scenario 4 (37.57%) and scenario 6 (56.07%), wherein the relative differences were closest to 0%. In contrast, scenarios 1, 3, and 5 demonstrated high relative differences (70 to 80%), thereby indicating a low similarity with the actual surgical results. In scenario 4, the y-axis translation, x-axis, and z-axis rotation were constrained, while the entire SC joint was constrained in scenario 6. Therefore, these two conditions were closer to reality than the other conditions.

### Comparison of Haller Index in chest wall shape after surgery and simulation

When CT scans reveal the cross-sectional shape of the chest wall, the expression and progression of the PE can be identified, and the surgical method can be determined. A numerical Haller index (HI) establishes the criteria for distinguishing between a normal chest wall and that of a patient with PE. This index is the ratio of the transverse diameter of the chest wall to the anterior-posterior diameter in the cross-sectional shape. This index can indicate a normal patient (HI < 3.25) or a patient with PE (HI > 3.25)^21,22^.

**Figure 6:**
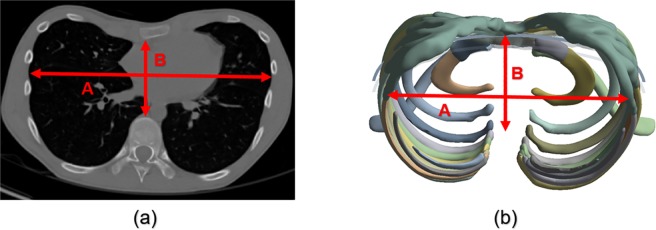
Measurement of HI from cross-sectional shape of chest wall from CT scan and simulation results: HI = A/B. (**a**) CT scan of preoperative patient; **(b)** Simulation result of scenario.

The posterior position of the anterior-posterior diameter of the HI should be specified as the end of the vertebral body in the inner chest wall. However, it was the end of the rib connected to the vertebrae that was specified in this FE model as it did not comprise a spine. The final anterior-posterior diameter was calculated by subtracting the length of the vertebrae. In this manner, postoperative and scenario simulation HIs were measured which confirmed whether the HI criteria (>3.25) were exceeded. In addition, the postoperative and scenario HIs were compared, and scenarios close to the actual postoperative HI were selected. Figure 6 shows the HI measurements obtained from the CT scan and simulation results. Table 6 shows the results of the measured HI values obtained postoperatively and from each scenario.

**Table 6:** Comparison of postoperative HI and those obtained from the various scenarios.

	Postoperative	Scenario Number					
		1	2	3	4	5	6
HI	2.672	2.626	2.633	2.625	2.683	2.606	2.686
Relative difference^a^(%)	0	1.72	1.64	1.75	0.41	2.47	0.52

^a^Quantification of the relative difference in HI for each scenario based on postoperative HI.

Table 6 shows that both the postoperative and simulation HI results did not exceed the normal criteria of 3.250, thereby indicating that all the types were within the normal range. Scenario 4 with HI = 2.683 and scenario 6 with HI = 2.686 can be selected as the scenarios closest to the postoperative HI based on relative differences. The SC joint conditions were similar to those of the previous sternal rotational angle study.

### Comparison of von-Mises equivalent stress on chest wall

**Figure 7:**
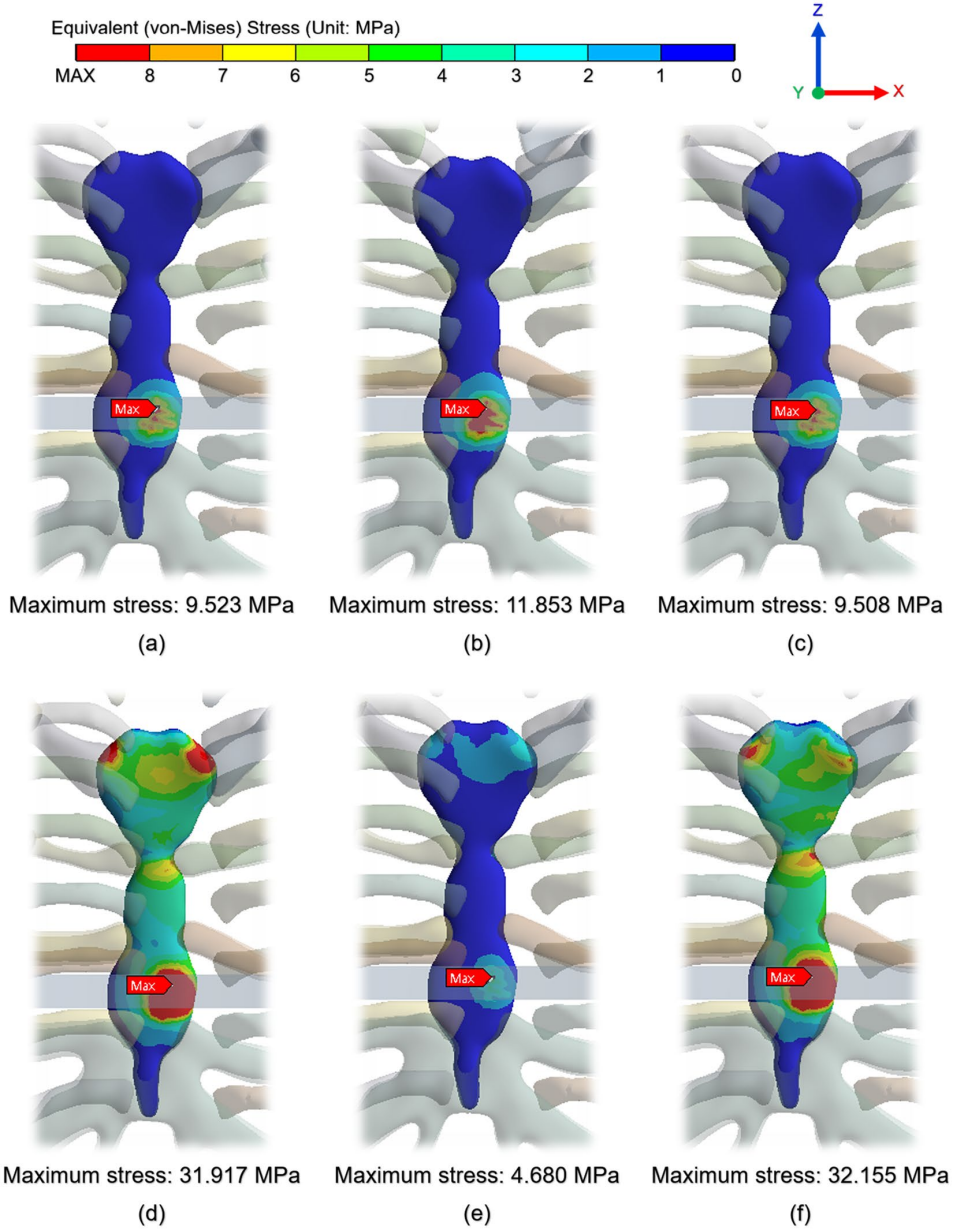
Equivalent stress distribution on chest wall and the point at which maximum equivalent stress is loaded by pectus bar: **(a)** Scenario 1; **(b)** Scenario 2; **(c)** Scenario 3; **(d)** Scenario 4; **(e)** Scenario 5; **(f)** Scenario 6.

As the displacement occurs on the chest wall, an equivalent stress is generated in each tissue and the pectus bar. The reality scenario can be identified from among all the scenarios, and the effect on the postoperative patient can be visualized by identifying the values of this physical force. Figures of the equivalent stress distribution on the chest wall were prepared, and the points at which the maximum equivalent stress was loaded in each scenario were identified. In Fig. 7, the analysis results show the stress distribution without the pectus bar in the model. The reason for this exclusion was that the equivalent stress on the pectus bar was determined to be insignificant.

Figure 7 shows that the equivalent stress was loaded to the sternum as a whole and was weakly transmitted to a part of the costal cartilage. Only those scenarios with y-axis translation constraints and all constraints produced significant stresses on the joints and sternum, with similar results being obtained for other conditions. The maximum equivalent stresses occurred mainly at the pectus bar and sternal contact points and were observed to be the same in all the scenarios. Moreover, the yield stress in the cortical bone of the sternum, ribs, and clavicle was approximately 70.0 MPa, and the costal cartilage was approximately 5.0 MPa^23,24^. In the above results, the maximum equivalent stress did not exceed the yield stress of each tissue, and the pectus bar also did not exceed the yield stress of titanium. This implies that the analysis performance conditions within the elastic section were suitably defined.

**Figure 8:**
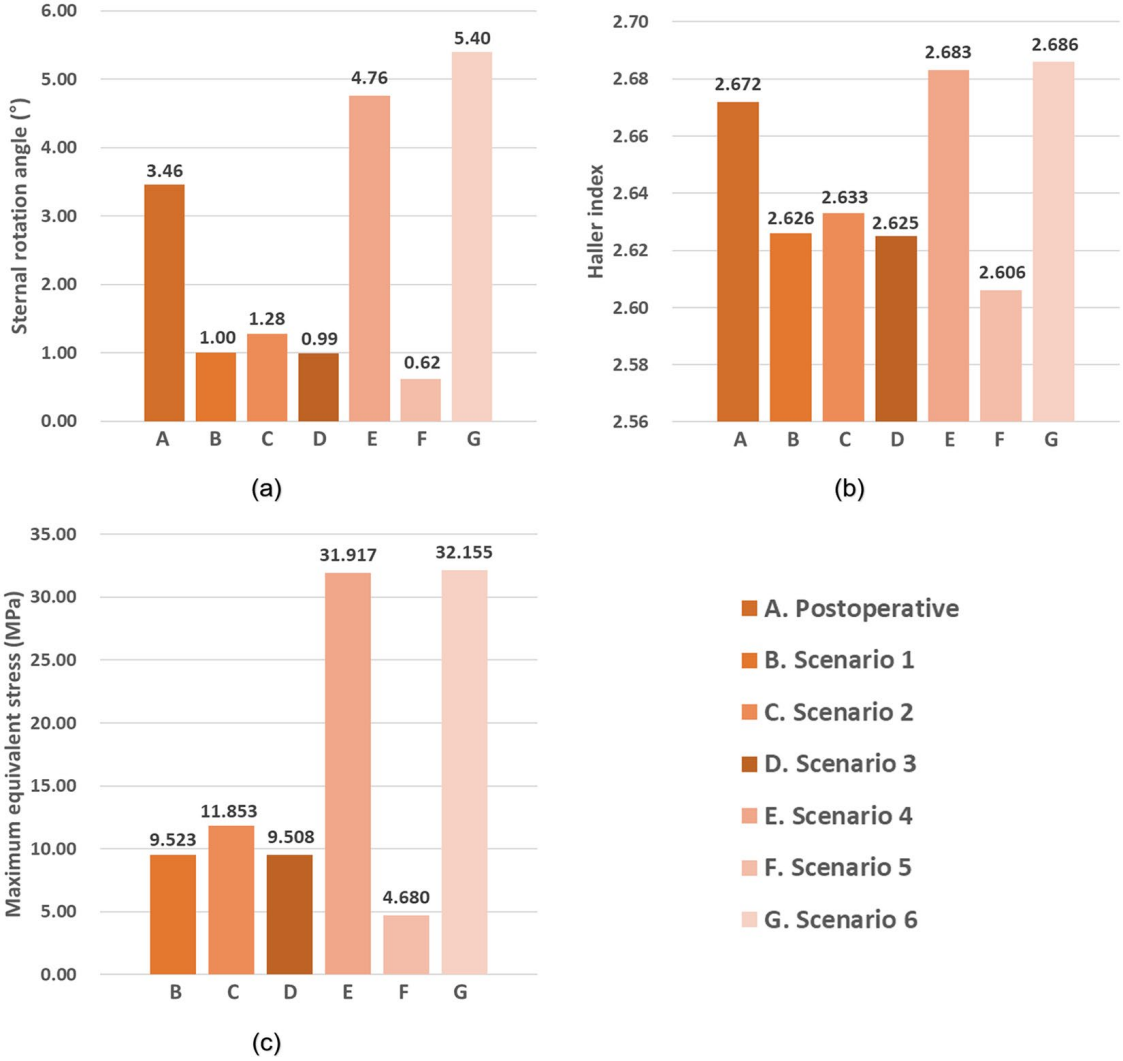
Comprehensive representation of postoperative results and for each scenario to confirm influence of SC joint. **(a)** Sternal rotational angle; **(b)** Haller index; **(c)** maximum equivalent stress.

As shown in Fig. 8, the graphs of each result can be divided into two groups based on trends. Scenarios 1, 2, 3, and 5 showed a sternal rotational angle of 0.6° to 1.3°, HIs of 2.60 to 2.64, and maximum stress of 4.7 to 11.9 MPa. Scenarios 4 and 6 showed a sternal rotational angle of 4.7° to 5.4°, HIs of 2.68 to 2.69, and a maximum stress of 31.9 to 32.2 MPa. The trends of the two groups differed significantly, and the groups in scenarios 4 and 6 were very similar to those as part of the postoperative results.

## Discussion

Each simulation was performed with a focus on the several scenarios classified according to the mechanical conditions of the SC joint. Thus, the sternal rotational angles, HI at the cross-sectional shape of the chest wall, and equivalent stresses on each tissue were determined. The data of the patients who completed the actual PE surgery and the simulation outputs were compared. The superiority and accuracy of the specific scenarios were analyzed, and the scenarios closest to the actual postoperative shape were selected. When compared to other conditions, scenarios 4 and 6 showed results that were more similar to the actual postoperative results. In particular, scenario 4 displayed the most similar results. Therefore, constraining the translation of the y-axis at the SC joints was found to yield the most similar behavior to that of the actual chest.

Several FE studies exist in which the Nuss procedure has been employed, but the majority of these did not consider the influence of the SC joints. For example, the influence of the SC joints on the 3D model of the chest wall or the constraints of analysis were not considered when an FE analysis was performed during application of a double bar or when suggesting a personalized design for a pectus bar in a detailed surgical method for PE patients^25,26^. In these studies, an FE analysis can more clearly demonstrate the superiority and advantage of the various personalized surgical methods by considering the constraints of the anterior-posterior translation of the SC joints.

An FE analysis related to previously published studies of the Nuss procedure has been performed to raise the sternum using a pectus bar. However, the pectus bar was inserted in a U shape at the insertion point of the chest wall in the actual Nuss procedure and rotated by 180° using a rotator^27^. Raising the sternum with the rotation of the bar restored the PE to its normal state. Even though the pectus bar is rotated during the actual surgery, most FE analysis studies, including this study, are only focused on the evaluation of the sternal raising. As the two movements are very different mechanically on the chest wall, it is necessary to simulate the rotational movement of the pectus bar in the FE analysis. Typically, patients exhibit various types of PEs based on the sternum’s deepest point and symmetry. In particular, when the deepest point or a long depression of the chest wall is corrected via the Nuss procedure, the influence of the SC joints, comprising the main resistance to sternal raising, is affected to a greater extent than other types. Therefore, the influence of the SC joints must be analyzed in these patients.

Biomaterials that constitute the chest wall exhibit complex characteristics such as elasto-plasticity, homogeneity, and failure. This study was simplified and only considered the elastic region of the material to identify the efficiency of the analyses. However, if the material properties can be applied more precisely, the accuracy of the results is expected to improve. In particular, studies focused on an analysis of the creep region may predict long-term results after chest wall surgery is performed. While the shapes of the costal cartilage can be precisely constructed, as in this study, considerable time is required for modelling, which complicates model development. Therefore, it is expected that more efficient studies could be conducted if the model can be simplified within a range that does not affect the overall mechanical behavior of the chest wall. The model was simplified for the convenience of FE analysis, and the study was performed while excluding conditions that did not affect the results. In addition to the tissues that were considered in this study, several muscles and ligaments constitute the chest wall. However, there is a limitation to the simplification without including them. This study also excludes several boundary conditions such as the mobility of costovertebral joints. A reduction in this range of simplification would be proportional to the accuracy of the results; thus, it is advisable to conduct further studies that consider various external conditions.

In this study, several scenarios were considered by mechanically dividing the movable areas of the SC joints. After setting the displacement and boundary conditions for each scenario, the results were analyzed via FE analysis. Thus, the influence of SC joints on the deformation of the chest wall during the actual surgery was quantified. It is proposed that the identified influences of the SC joints should be considered in simulations via usage of details such as the number, fixation, and position of the pectus bars in the Nuss procedure. To overcome the various limitations, such as the rotational movement of the pectus bar, analysis of patients with various types of PEs, and accuracy of the properties of the materials, there is a need to establish actionable measures. In future studies, it is expected that the influence of the SC joints will be clearer and more defined than those identified in this study, which is expected to further enhance technical developments in FE analyses of the chest wall.

The SC joints were assessed based on the degree to which they affected the results of various chest wall simulations. Various types of SC joint behaviors were defined from a mechanical viewpoint, and the obtained results of the simulations indicated that the translation in the anterior–posterior direction of the chest wall was more important than other types of movements. In addition, it was more realistic to replicate these SC joint conditions while comparing the results with cases of actual surgical patients.

## Conclusion

These results suggest that the influence of SC joints should be considered to obtain more accurate FE analyses of chest walls. It is also expected that this study could provide clinicians with more accurate mechanical results and patient-specific information in terms of preoperative chest wall predictions.

## Supplementary information

Supplementary Table S1.
