# Supplementary material for: Numerical investigation of the sternoclavicular joint modeling technique for improving the surgical treatment of pectus excavatum
Source: Sci Rep. 2020 Apr 30;10:7357. doi: 10.1038/s41598-020-64482-7 (PMC7192908; doi:10.1038/s41598-020-64482-7)
Supplement: Supplementary file 1 — Supplementary Table S1. [file 41598_2020_64482_MOESM1_ESM.docx]

**Numerical investigation of the sternoclavicular joint modeling technique for improving the surgical treatment of pectus excavatum**

# Beop-Yong Lim^1,2^, Youngwoong Kim^3^, Hoseok I^4,5,*^, and Chi-Seung Lee^5,6,7,*^

^1^Department of Biomedical Engineering, Gradate School, Pusan National University, Busan 49241, Republic of Korea

^2^University Research Park of Pusan National University, Busan 49241, Republic of Korea

^3^Department of Thoracic and Cardiovascular Surgery, Trauma Center, Ulsan University Hospital, University of Ulsan College of Medicine, Ulsan 44033, Republic of Korea

^4^Department of Thoracic and Cardiovascular Surgery, School of Medicine, Pusan National University, Busan 49241, Republic of Korea

^5^Biomedical Research Institute, Pusan National University Hospital, Busan 49241, Republic of Korea

^6^Department of Convergence Medicine, School of Medicine, Pusan National University, Busan 49241, Republic of Korea

^7^Department of Biomedical Engineering, School of Medicine, Pusan National University, Busan 49241, Republic of Korea

^*^corresponding authors: Hoseok I and Chi-Seung Lee; ihoseok@pusan.ac.kr and victorich@pusan.ac.kr

The material properties of the clavicle were simplified, and the reason was that there was little influence of these properties in the finite element analysis of the chest wall. We conducted a comparative analysis of the clavicle properties and the results were as table S1.

|  | Cortical bone | Cortical and cancellous bone | Difference |
| --- | --- | --- | --- |
| Rotation angle (°) | 1.28 | 1.36 | 0.08 |
| Haller index | 2.633 | 2.635 | 0.002 |
| Equivalent stress (MPa) | 11.853 | 12.445 | 0.592 |

**Supplementary Table S1.** Comparative finite element analysis according to clavicle material properties
